# *Streptomyces asenjonii* sp. nov., isolated from hyper-arid Atacama Desert soils and emended description of *Streptomyces viridosporus* Pridham et al. 1958

**DOI:** 10.1007/s10482-017-0886-7

**Published:** 2017-06-06

**Authors:** Michael Goodfellow, Kanungnid Busarakam, Hamidah Idris, David P. Labeda, Imen Nouioui, Roselyn Brown, Byung-Yong Kim, Maria del Carmen Montero-Calasanz, Barbara A. Andrews, Alan T. Bull

**Affiliations:** 1 School of Biology, Newcastle University, Ridley Building 2, Newcastle upon Tyne, NE1 7RU UK; 2 National Centre for Agricultural Utilization Research, USDA ARS, Peoria, IL 61614 USA; 3 Chunlab Inc., Seoul Natural University, Gwanak-ro, Gwanak-gu, Seoul, 151-742 Republic of Korea; 4 School of Biosciences, University of Kent, Canterbury, Kent CT2 7NJ UK; 5 Centre for Biotechnology and Bioengineering (CeBiB), Department of Chemical Engineering and Biotechnology, University of Chile, Beauchef, 851 Santiago, Chile

**Keywords:** *Streptomyces*, Polyphasic taxonomy, Hyper-arid, Atacama Desert

## Abstract

A polyphasic study was undertaken to establish the taxonomic status of *Streptomyces* strains isolated from hyper-arid Atacama Desert soils. Analysis of the 16S rRNA gene sequences of the isolates showed that they formed a well-defined lineage that was loosely associated with the type strains of several *Streptomyces* species. Multi-locus sequence analysis based on five housekeeping gene alleles showed that the strains form a homogeneous taxon that is closely related to the type strains of *Streptomyces ghanaensis* and *Streptomyces viridosporus*. Representative isolates were shown to have chemotaxonomic and morphological properties consistent with their classification in the genus *Streptomyces.* The isolates have many phenotypic features in common, some of which distinguish them from *S. ghanaensis* NRRL B-12104^T^, their near phylogenetic neighbour. On the basis of these genotypic and phenotypic data it is proposed that the isolates be recognised as a new species within the genus *Streptomyces*, named *Streptomyces asenjonii* sp. nov. The type strain of the species is KNN35.1b^T^ (NCIMB 15082^T^ = NRRL B-65050^T^). Some of the isolates, including the type strain, showed antibacterial activity in standard plug assays. In addition, MLSA, average nucleotide identity and phenotypic data show that the type strains of S. *ghanaensis* and *S. viridosporus* belong to the same species. Consequently, it is proposed that the former be recognised as a heterotypic synonym of the latter and an emended description is given for *S. viridosporus*.

**Electronic supplementary material:**

The online version of this article (doi:10.1007/s10482-017-0886-7) contains supplementary material, which is available to authorized users.

## Introduction

The prospect of isolating novel filamentous actinobacteria that synthesise new specialised metabolites is enhanced when bioprospecting strategies are focused on neglected and unexplored habitats (Hong et al. 2009; Tiwari and Gupta 2012; Guo et al. 2015), including desert soils (Meklat et al. 2011; Boubetra et al. 2013). The most extensive surveys of culturable actinobacterial diversity in desert biomes have been concentrated on sites in the Atacama Desert in northern Chile, the driest non-polar desert on the planet (Bull and Asenjo 2013; Bull et al. 2016). The application of a taxonomic approach to drug discovery (Goodfellow and Fiedler 2010) has been effective in the isolation of putatively novel filamentous actinobacteria from Atacama Desert habitats, some of which produce novel natural products (Bull et al. 2016; Wichner et al. 2016). Indeed, polyphasic taxonomic studies on dereplicated actinobacteria isolated from hyper-arid and extreme hyper-arid Atacama Desert soils have led to the description of novel species of *Lechevalieria* (Okoro et al. 2010), *Lentzea* (Idris et al. 2017a) and *Modestobacter* (Busarakam et al. 2016a) and to the detection of rare thermophilic *Amycolatopsis* species (Busarakam et al. 2016b). In addition, several new *Streptomyces* species have been described (Santhanam et al. 2012a, b, 2013; Idris et al. 2017b), one of which, *Streptomyces leeuwenhoekii* (Busarakam et al. 2014), encompasses strains that synthesise novel antibiotics (Nachtigall et al. 2011; Rateb et al. 2011a, b) and chaxapeptin, a new lasso peptide (Elsayed et al. 2015).

The present study was designed to establish the taxonomic position of several closely related Atacama Desert streptomycetes. These strains were the subject of a polyphasic taxonomic study which showed that they belong to a new species, *Streptomyces asenjonii* sp. nov.

## Materials and methods

### Isolation, maintenance and cultivation of strains

Isolates KNN6.11a, KNN35.1b^T^, KNN35.2b, KNN48.3e and KNN83.e were recovered from a hyper-arid soil collected in 2012 by one of us (ATB) from the Chaxa de Laguna, Salar de Atacama near Tocanão (23°17′33″S, 68°10′99″W at 2219 m above sea level), using the dilution plate procedure described by Okoro et al. (2009). The strains were isolated on Gauze’s No.1 agar (KNN6.11a) (Zakharova et al. 2003), humic acid-vitamin agar (KNN35.1b^T^, KNN35.2b) (Hayakawa and Nonomura 1987) and SM1 agar (KNN48.3e, KNN83.e) (Tan et al. 2006) following incubation for 14 days at 28 °C. Similarly, the final strain, KNN42.f, was isolated from a starch-casein agar plate (Küster and Williams 1964) following inoculation with a suspension of an extreme hyper-arid soil collected by ATB in 2010 from the Yungay core region of the Atacama Desert (24°06′18.6″S, 70°01′55.6″W at 1016 m asl). These strains, together with *Streptomyces ghanaensis* NRRL B12104^T^ (Wallhäuser et al. 1965), were maintained on yeast extract—malt extract agar (International *Streptomyces* Project [ISP2] medium., Shirling and Gottlieb 1966) and as suspensions of spores and hyphal fragments in 20%, v/v glycerol at −20 and −80 °C. Biomass samples for most of the chemotaxonomic analyses and for the 16S rRNA gene sequencing studies were prepared in shake flasks (180 revolutions per minute) of ISP 2 broth after incubation at 28 °C for 14 days and washed twice in distilled water. Cells for the chemotaxonomic analyses were freeze-dried and those for the sequencing studies stored at room temperature. Biomass preparations for the fatty acid analyses were harvested from shake flasks of Tryptic Soy broth (Difco) following incubation at 28 °C for 7 days.

### Phylogenetic analysis

16S rRNA gene sequencing. Genomic DNA extraction, PCR-mediated amplification of 16S rRNA genes and purification of the resultant products were carried out on all of the isolates using the procedures described by Kim and Goodfellow (2002). Identification of phylogenetic neighbours and calculation of pairwise 16S rRNA gene sequence similarities were achieved using the EzTaxon-e server (http://www.ezbiocloud.net/taxonomy; Yoon et al. 2017) and the resultant sequences aligned using the CLUSTAL W algorithm from the MEGA 6 software package (Tamura et al. 2013). Phylogenetic analyses using the maximum-likelihood (ML) (Felsenstein 1981) and maximum-parsimony (MP) algorithms (Fitch 1971) were also realised using the GGDC web server (Meier-Kolthoff et al. 2013a) of the DSMZ phylogenomics pipeline (Meier-Kolthoff et al. 2014) adapted to single genes available at http://ggdc.dsmz.de/. ML and MP trees were inferred from the alignment with RAxML (Stamatakis 2014) and TNT (Goloboff et al. 2008), respectively. The topologies of the resultant trees were evaluated by bootstrap analyses (Felsenstein 1985) based on 1000 replicates used in conjunction with tree-bisection-and-reconnection branch swapping and ten additional random sequence replicates for MP and rapid bootstrapping in conjunction with the auto MRE bootstopping criterion (Pattengale et al. 2010) for ML. The trees were rooted using the 16S rRNA gene sequence of *Streptomyces albus* subspecies *albus* DSM 40317^T^ (GenBank accession number AJ621602). The Χ^2^ test implemented in PAUP* (Swofford 2002) was used to check for compositional bias. Pairwise sequence similarities were calculated using the method recommended by Meier-Kolthoff et al. (2013b) for 16S rRNA genes and a multiple sequence alignment was created with MUSCLE (Edgar 2004).

Multi-locus sequence analysis. Genomic DNA extracted from each of the isolates following growth in ISP2 broth at 28 °C was purified, as described by Idris et al. (2017a). The housekeeping genes used in previous analyses on streptomycetes (Busarakam et al. 2014; Labeda et al. 2017; Idris et al. 2017b; Labeda 2016), namely *atpD* (ATP synthase F1, beta subunit), *gyrB* (DNA *gyrB* subunit), *rpoB* (RNA polymerase beta subunit), *recA* (recombinase A) and *trpB* (tryptophane B, beta subunit), were amplified, sequenced, purified, deposited in the GenBank database and organised using the Bacterial Isolate Genome Sequence Database BIGSdb version 1.15.4 on the ARS Microbial Genome Sequence Database server (http://199.133.98.43). The sequences of the protein loci of the strains were aligned with one another and with those of their close neighbours and phylogenetic relationships established using the ML algorithm after Idris et al. (2017a). Pairwise distances between the sequences of each locus were established using the Kimura two-parameter model (Kimura 1980). Strain pairs having MLSA evolutionary distances ≤0.007 were considered conspecific based on the cut-off point empirically determined by Rong and Huang (2012, 2014), a value that corresponds to the 70% DNA:DNA threshold recommended for the delineation of prokaryotic species by Wayne et al. (1987).

### Draft genome preparation and ANI calculations

The draft genome sequence of *Streptomyces viridosporus* NRRL 2414^T^ was prepared following the protocol outlined in Labeda et al. (2016) with the exception that CLCbio Genomic Workbench Version 9.5.3 (CLCbio; Boston, MA) was used for contig trimming and *de novo* assembly. This Whole Genome Shotgun project has been deposited at DDBJ/EMBL/GenBank under the accession MSGP00000000.

The draft genome sequence of NRRL 2414^T^ was compared with the draft genomes sequences of *S. viridosporus* T7A (Genbank accession number AJFD00000000), *S. ghanaensis* ATCC 14672^T^ (GenBank accession number ABYA00000000), *Streptomyces hirsutus* NRRL B-3713^T^ (GenBank accession number LIQT00000000), and *Streptomyces cyanoalbus* NRRL B-3040^T^ (GenBank accession number LIPS00000000) obtained from Genbank utilising the calculate_ani.py script (https://github.com/widdowquinn/sripts/blob/master/bioinformatics/calculate_ani.py) which implements the methods described by Goris et al. (2007) and Richter and Rosselló-Móra (2009), with results shown in Supplemental Table S1.

### Chemotaxonomy and morphology

Isolates KNN35.1b^T^ and KNN35.2b were examined for spore chain arrangement and spore-surface ornamentation following growth on oatmeal agar (ISP 3 medium; Shirling and Gottlieb 1966) for 14 days at 28 °C, by scanning electron microscopy (Cambridge 240 instrument), using the protocol described by O’Donnell et al. (1993). Key chemotaxonomic markers were sought using standard chromatographic procedures. All of the isolates were examined for isomers of diaminopimelic acid (A_2_pm) after Staneck and Roberts (1974). Strains KNN35.1b^T^ and KNN35.2b were analysed for menaquinone, whole cell sugar and polar lipid composition using the procedures described by Collins et al. (1985), Lechevalier and Lechevalier (1970) and Minnikin et al. (1984), respectively. *S. ghanaensis* NRRL B-12104^T^, the close phylogenetic neighbour of the isolates, was included in the sugar and polar lipid analyses. Fatty acids of representative isolates, namely strains KNN35.1b^T^, KNN35.2b and KNN42.f, and the *S. ghanaensis* type strain, were extracted, methylated and analysed using the established Sherlock Microbial Identification (MIDI) system and the ACTIN version 6 database (Sasser 1990).

### Cultural characteristics

The cultural properties of the isolates were recorded on tryptone-yeast extract, yeast extract-malt extract, inorganic salts-starch, glycerol-asparagine, peptone-yeast extract-iron and tyrosine agar plates (ISP media 1-7, Shirling and Gottlieb 1966) after 14 days at 28 °C. Aerial and substrate mycelial colours and those of diffusible pigments were determined by comparison against chips from the Inter-Society Colour Council-National Bureau of Standard Colour charts (Kelly 1964).

### Phenotypic tests

The isolates and *S. ghanaensis* NRRL B-12104^T^ were examined for standard biochemical, degradative and physiological characteristics after Williams et al. (1983) and enzyme profiles determined using API-ZYM kits (BioMerieux) employing a standardised inoculum corresponding to 5 on the McFarland scale (Murray et al. 1999) and the protocol provided by the manufacturer. The oxidation of carbon sources and resistance to inhibitory compounds were determined using GENIII microplates in an Omnilog device (Biolog Inc., Haywood, USA). The microplates were inoculated with cell suspensions made in a ‘gelling’ inoculating fluid at a cell density of 98% transmittance with a run time of 7 days in phenotypic microarray mode at 28 °C. The exported data were analysed using the opm package for R version 1.0.6. (Vaas et al. 2012, 2013). The Biolog tests were carried out in duplicate.

### Antibacterial sensitivity assays

Four of the isolates, strains KNN6.11a, KNN35.1b^T^, KNN35.2b and KNN83.e, were examined for their ability to inhibit the growth of wild type strains of *Bacillus subtilis, Escherichia coli, Pseudomonas fluorescens* and *Staphylococcus aureus* using a standard plug assay (Fiedler 2004). The isolates were grown on yeast extract-malt extract sloppy agar (0.8%, w/v agar) for 14 days at 30 °C and then plugs were transferred to nutrient agar plates which had been inoculated with 100 µl of the wild type strains grown overnight in lysogeny broth. The inoculated plates were incubated overnight and then examined for the presence of inhibition zones around the agar plugs.

## Results and discussion

**Fig. 1:**
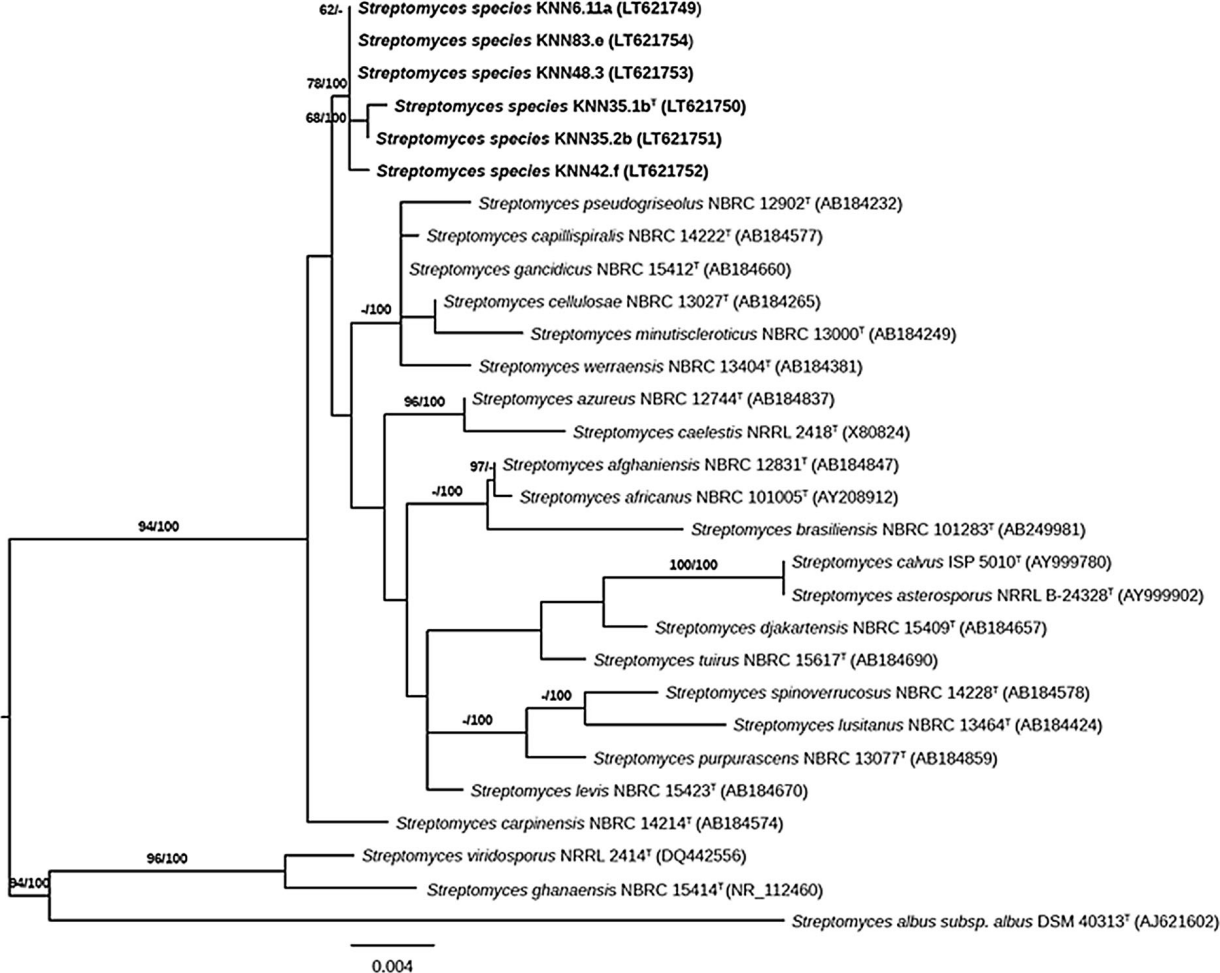
Maximum-likelihood phylogenetic tree based on 16S rRNA sequences showing relationships between isolates KNN6.11a, KNN 35.1b, KNN 35.2b, KNN 42.f, KNN 48.3, and KNN83.e and between them and the type strains of the most closely related *Streptomyces* species, the tree was inferred using the GTR+GAMMA model. The branches are scaled in terms of the expected number of substitutions per site. The numbers above the branches are support values when larger than 60% from ML (*left*) and MP (*right*) bootstrapping

The six strains isolated from the hyper-arid and extreme hyper-arid Atacama Desert soils were shown to form a well delineated subclade in the *Streptomyces* 16S rRNA gene tree, a relationship that was supported by all of the tree-making algorithms and by a 78% bootstrap value (Fig. 1). The isolates were found to share 16S rRNA gene sequence similarities within the range 99.85–100%, which corresponds to up to 3 nucleotide (nt) differences at 1373 locations. The strains were seen to be closely related to the type strains of *Streptomyces gancidicus* DSM 40935 (99.57–99.64% similarity), *Streptomyces pseudogriseolus* DSM 40026^T^ (99.49–99.58% similarity), *Streptomyces capillispiralis* DSM 41695^T^ (99.49–99.57% similarity), *Streptomyces werraensis* DSM 40486^T^ (99.34–99.51% similarity), *Streptomyces minutiscleroticus* DSM 40301^T^ (99.05–99.15% similarity) and *Streptomyces cellulosae* DSM 40362^T^ (99.35–99.50% similarity). These data suggest that the Atacama Desert isolates are not particularly closely related to any of their near phylogenetic neighbours in the *Streptomyces* 16S rRNA gene tree.

**Fig. 2:**
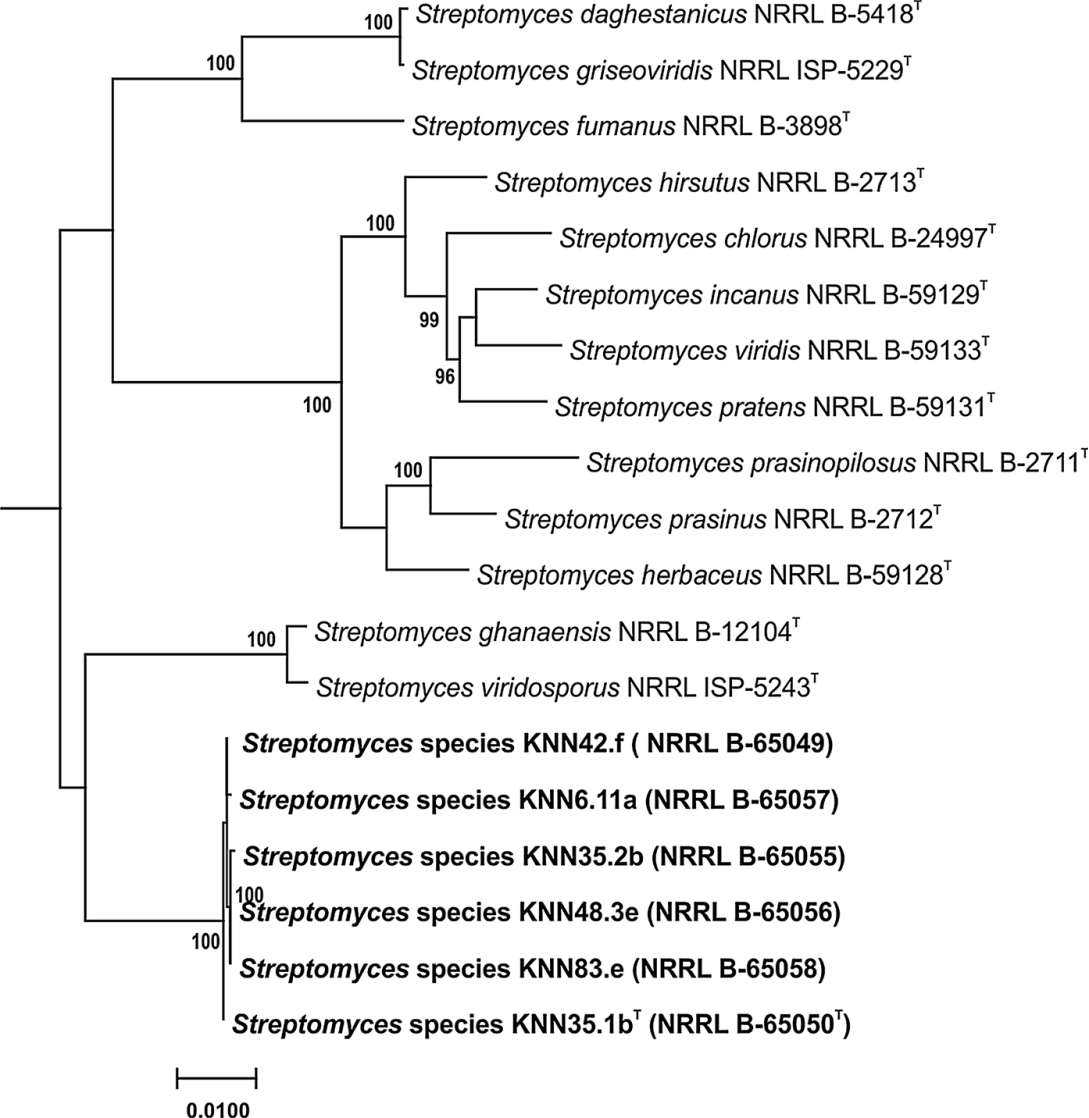
Subtree from the *Streptomyces* phylogenetic tree inferred from concatenated partial sequences of the house-keeping genes *atpD, gyrB, recA, rpoB* and *trpB* in IQ-Tree version 1.4.2 (Nguyen et al. 2015) as described by Labeda et al. (2017). Bootstrap values less than 95% were omitted as suggested by the IQ-Tree developers. *Bar scale* reflects number of substitutions per site

The isolates were found to belong to a distinct and homogeneous lineage in the *Streptomyces* MLSA gene tree based on concatenated partial sequences of the five housekeeping genes, a result supported by a 100% bootstrap value (Fig. 2). The MLSA evolutionary distances between the isolates ranged from <0.000 to 0.001 (Table 1), that is, well within the species level threshold of ≤0.007 proposed by Rong and Huang (2012, 2014). Members of this well delineated taxon were found to be closely related to the type strains of *S. ghanaensis* DSM 40746^T^ and *S. viridosporus* DSM 40243^T^ (Pridham et al. 1958), albeit with MLSA distances well above the species cut-off point (Table 1). These results provide further evidence of the value of MLSA sequence analyses in clarifying the subgeneric relationships of *Streptomyces* (Guo et al. 2008; Rong and Huang 2010, 2012, 2014; Busarakam et al. 2014; Idris et al. 2017b; Labeda et al. 2014, 2017; Labeda 2016). The *S. ghanaensis* and *S. viridosporus* strains formed a well-supported subclade in the 16S rRNA gene tree (Fig. 1) but were not particularly closely related (99.5% sequence similarity, 19 nt differences), results that are clearly more apparent than real given the corresponding MLSA data.

**Table 1:** MLSA distances calculated for species phylogenetically near to the proposed new species, *Streptomyces asenjonii*

	MLSA (Kimura 2-parameter) distance								
*S. daghestanicus* NRRL B-5418^T^	–								
*S. griseoviridis* NRRL ISP-5229^T^	**0.001**	–							
*S. fumanus* NRRL B-3898^T^	0.034	0.034	–						
*S. hirsutus* NRRL B-2713^T^	0.060	0.060	0.062	–					
*S. chlorus* NRRL B-24997^T^	0.064	0.063	0.064	0.024	–				
*S. incanus* NRRL B-59129^T^	0.063	0.063	0.060	0.021	0.019	–			
*S. viridis* NRRL B-59133^T^	0.063	0.063	0.062	0.016	0.021	0.016	–		
*S. pratens* NRRL B-59131^T^	0.063	0.063	0.065	0.023	0.021	0.016	0.021	–	
*S. prasinopilosus* NRRL B-2711^T^	0.058	0.058	0.058	0.039	0.038	0.040	0.043	0.041	–
*S. herbaceous* NRRL B-59128^T^	0.060	0.060	0.058	0.029	0.032	0.026	0.031	0.031	0.027
*S. prasinus* NRRL B-2712^T^	0.058	0.058	0.056	0.030	0.035	0.036	0.035	0.036	0.023
*S. ghanaensis* NRRL B-12104^T^	0.052	0.051	0.052	0.052	0.056	0.057	0.054	0.060	0.059
*S. viridosporus* NRRL ISP-5243^T^	0.051	0.051	0.053	0.050	0.055	0.056	0.054	0.058	0.057
*S.* species KNN35.1b^T^ (NRRL B-65050^T^)	0.043	0.043	0.043	0.051	0.055	0.054	0.051	0.058	0.055
*S.* species KNN42.f (NRRL B-65049)	0.044	0.043	0.043	0.051	0.055	0.055	0.052	0.059	0.055
*S.* species KNN35.2b (NRRL B-65055)	0.045	0.044	0.044	0.052	0.056	0.056	0.053	0.060	0.056
*S.* species KNN48.3e (NRRL B-65056)	0.044	0.044	0.043	0.051	0.056	0.055	0.052	0.059	0.056
*S.* species KNN6.11a (NRRL B-65057)	0.044	0.044	0.043	0.051	0.056	0.055	0.052	0.059	0.056
*S.* species KNN83.e (NRRL B-65058)	0.044	0.044	0.043	0.051	0.056	0.055	0.052	0.059	0.056

	MLSA (Kimura 2-parameter) distance								
*S. daghestanicus* NRRL B-5418^T^									
*S. griseoviridis* NRRL ISP-5229^T^									
*S. fumanus* NRRL B-3898^T^									
*S. hirsutus* NRRL B-2713^T^									
*S. chlorus* NRRL B-24997^T^									
*S. incanus* NRRL B-59129^T^									
*S. viridis* NRRL B-59133^T^									
*S. pratens* NRRL B-59131^T^									
*S. prasinopilosus* NRRL B-2711^T^									
*S. herbaceous* NRRL B-59128^T^	–								
*S. prasinus* NRRL B-2712^T^	0.020	–							
*S. ghanaensis* NRRL B-12104^T^	0.056	0.053	–						
*S. viridosporus* NRRL ISP-5243^T^	0.054	0.052	**0.004**	–					
*S.* species KNN35.1b^T^ (NRRL B-65050^T^)	0.054	0.051	0.036	0.037	–				
*S.* species KNN42.f (NRRL B-65049)	0.054	0.052	0.036	0.037	**0.000**	–			
*S.* species KNN35.2b (NRRL B-65055)	0.056	0.053	0.037	0.038	**0.001**	**0.001**	–		
*S.* species KNN48.3e (NRRL B-65056)	0.056	0.052	0.036	0.038	**0.000**	**0.001**	**0.000**	–	
*S.* species KNN6.11a (NRRL B-65057)	0.056	0.052	0.036	0.038	**0.000**	**0.001**	**0.001**	**0.001**	–
*S.* species KNN83.e (NRRL B-65058)	0.056	0.052	0.036	0.038	**0.000**	**0.001**	**0.000**	**0.000**	**0.001**

**Fig. 3:**
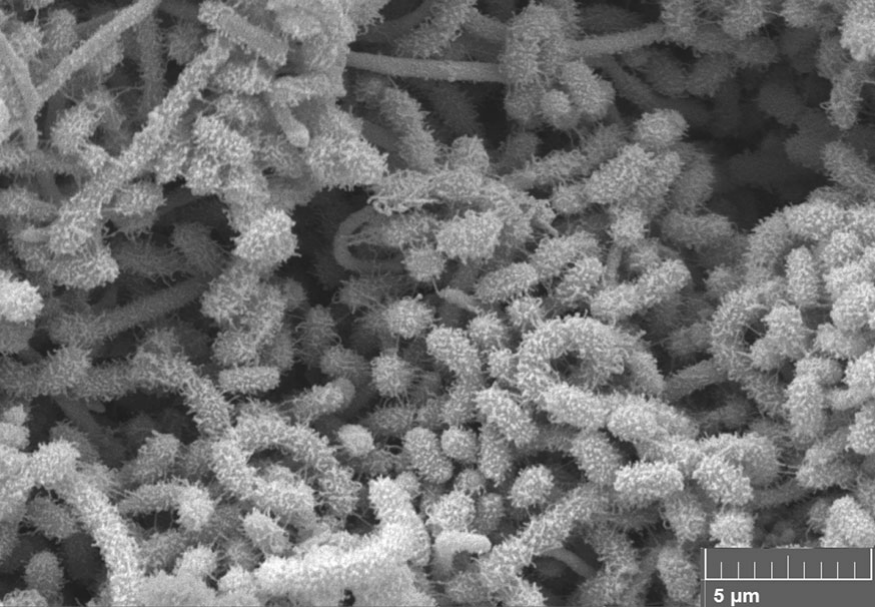
Scanning electron micrograph of isolate KNN35-1b^T^ showing hairy ornamented spores in open spirals following growth on oatmeal agar at 28 °C for 14 days. *Bar* 1 µm

The isolates were shown to form extensively branched substrate mycelia bearing aerial hyphae, to contain LL-A_2_pm as the wall diamino acid and exhibited good growth on all of the ISP media, notably on oatmeal and yeast extract-malt extract agar (Table 2). In general, the substrate mycelia were observed to be grey to yellowish white and the aerial spore mass greyish yellow or bright orange yellow, as were the diffusible pigments. Isolates KNN35.1b^T^ and KNN35.2b were seen to form open spirals of hairy ornamented surfaced spores, as shown in Fig. 3. These isolates and *S. ghanaensis* NRRL B-12104^T^, their close phylogenetic neighbour, were found to have glucose, mannose, ribose and xylose in whole organism hydrolysates, whilst the *S. ghanaensis* strain was also found to contain galactose. The polar lipid patterns of these strains showed the presence of diphosphatidylglycerol, glycophospholipid, phosphatidylethanolamine, phosphatidylglycerol, phosphatidylinositol and a number of unidentified components, as shown in Figure S1. The predominant isoprenologs seen in isolates KNN35.1b and KNN35.2b^T^ were identified as MK9 (H_6_) (~35%), MK9 (H_8_) (~30%) and MK9 (H_4_) (~10%). All of these properties are consistent with the classification of the isolates in the genus *Streptomyces* (Kämpfer 2012; Idris et al. 2017b). Complex mixtures of saturated and branched chain fatty acids were found in the representative isolates and in the type strain of *S. ghanaensis* (Table 3). The predominant components in all of these organisms were found to be *anteiso*–C_15:0_ (11.5–17.8%) and *iso*–C_16:0_ (21.3–32.6%); quantitative differences were seen in these and other components while some of the minor fatty acids were discontinuously distributed, as exemplified by the presence of *anteiso*-C_17:1_ and C_17:1_ *cis*9 in the *S. ghanaensis* type strain and *anteiso*-C_18:0_ amongst the isolates.

**Table 2:** Growth and cultural characteristics of all of the isolates on ISP media after incubation for 14 days at 28 °C

Media	Growth	Substrate mycelium colour	Aerial spore mass colour	Diffusible pigment
Glycerol-asparagine agar (ISP 5)	+++	Dark grey	Dark grey	None
Inorganic salts-starch agar (ISP 4)	+++	Yellowish white	Light yellowish orange	Light yellowish orange
Oatmeal agar (ISP 3)	++++	Yellowish white	Light yellowish orange	Light yellowish orange
Peptone-yeast extract-iron agar (ISP 6)	+++	Yellowish grey	Olivaceous grey green	Yellowish grey
Tryptone-yeast extract agar (ISP1^a^)	+++	Yellowish white	Light yellowish orange	Light yellowish orange
Tyrosine agar (ISP 7)	+++	Yellowish white	Light yellowish orange	Light yellowish orange
Yeast extract-malt extract agar (ISP 2)	++++	White	Dark yellowish orange	Yellowish grey

++++ abundant growth; +++ very good growth

^a^ISP1 agar medium

**Table 3:** Fatty acid profiles (%) of representatives *Streptomyces* isolates and the type strain of *S. ghanaensis*

Fatty acids	Isolate KNN 35.1b^T^	Isolate KNN 35.2b	Isolate KNN 42.f	*S. ghanaensis* NRRL B-12104^T^
*Iso*-C_14:0_	5.3	7.6	8.1	3.7
*Anteiso*- C_15:0_	17.5	15.1	17.8	11.5
*Iso*-C_15:0_	11.3	7.2	8.4	5.2
C_16:0_	4.0	–	6.7	3.0
*Iso*-C_16:0_	24.2	21.3	28.6	32.6
*Iso*- H C_16:0_	3.0	8.3	–	–
C_16:0_	–	8.8	–	3.0
*Iso*-H C_16:1_	–	–	4.4	8.8
*Iso*-C_17:0_	6.9	3.4	3.7	2.7
*Anteiso*- C_17:0_	9.9	6.4	7.8	12.3
C_17:1_ CIS 9	–	–	–	1.2
C_17:1_ ω 8c	0.9	–	0.7	1.2
*Anteiso*- C_17:1_ ω 9c	3.4	3.3	3.7	6.6
*Anteiso* –C_17:1_	–	–	–	6.6
C_17:0_	0.8	–	0.9	0.4
C_17:0_ 10-methyl	–	–	–	0.2
C_18:0_	0.3	4.2	1.3	–
C_18:0_ ω 9c	–	–	–	–
*Iso*- H C_18:1_	0.9	–	–	1.4
Summed feature 3	2.0	3.3	2.8	4.0
Summed feature 5		6.1	1.7	–
Summed feature 9	6.6	5.1	3.0	3.8

Trace proportions (<0.9%) are only cited for strains where other fatty acids were found at levels beyond this cut-off point

Summed feature 3, C_16:1_ ω7cand/orC_16:1_ ω6c; summed feature 5, *iso*-C_17:1_ ω9c and/or C_18:2_ ω6,9c; summed feature 9, *iso*-C_19_ ω8a and/or *iso*-C_17:1_ ω9c

Identical results were obtained for nearly all of the duplicated strains included in the phenotypic tests, whilst the exceptions were a few of the carbon source features recorded from the GENIII microplates (Table 4). It can also be seen from Table 4 that the isolates can be distinguished from one another showing that they are not clones. In addition, several properties distinguished all of the isolates from the type strain of *S. ghanaensis* (Table 4). Thus, only the Atacama isolates produced *N*-acetyl-β-glucosaminidase, oxidised l-arginine, butyric acid, l-*keto*-butryric acid, citric acid, d-and l-fucose and d-sorbitol and grew in the presence of 4%, w/v sodium chloride, potassium tellurite and rapamycin SV and at 10 °C. In contrast, only *S. ghanaensis* NRRL B-12104^T^ oxidised *N*-acetyl-β-d-mannosamine, *N*-acetyl-neuraminic acid and d-glucuronic acid. It is also apparent from Table 4 that all of the strains have many phenotypic properties in common.

**Table 4:** Phenotypic tests that distinguish the isolates from one another and from *Streptomyces ghanaensis* NRRL B-12104^T^

Characteristic	Isolate KNN 6.11a	Isolate KNN 35.1b^T^	Isolate KNN 35.2b	Isolate KNN 42.f	Isolate KNN 43.e	Isolate KNN 83.e	*S. ghanaensis* NRRL B-12104^T^
API ZYM tests							
*N*-Acetyl-β-glucosaminidase	+	+	+	+	+	+	–
Esterase (C4)	+	–	–	–	–	+	–
α-Glucuronidase	+	–	+	–	–	+	+
α-Mannosidase	–	+	–	+	–	–	+
GEN III BIOLOG microplate tests							
(a) Oxidation of sugars							
*N*-acetyl-D-galactosamine	+	–	–	–	–	+	+
*N*-acetyl-β-d-mannosamine	–	–	–	–	–	–	+
*N*-acetyl-neuraminic acid	–	–	–	–	–	–	+
d-Fucose	+	+	+	+	+	+	–
l-Fucose	+	+	+	+	+	+	–
d-Glucose-6-phosphate	–	+	–	+	–	–	+
α-d-Lactose	+	+	+	–	+	–	+
d-Mannitol	+	+	+	–	+	–	+
β-methyl-d-Glucoside	+	+	–	–	–	–	–
d-Salicin	+	–	–	–	–	+	+
d-Sorbitol	+	+	+	+	+	+	–
(b) Oxidation of amino acids							
l-Arginine	+	+	+	+	+	+	–
l-Serine	+	+	–	+	–	–	+
(c) Oxidation of organic acids							
Bromo-succinic acid	+	+	–	–	–	–	–
Butyric acid	+	+	+	+	+	+	–
α-*keto*-Butyric acid	+	+	+	+	+	+	–
Citric acid	+	+	+	+	+	+	–
α-*keto*-Glutaric acid	+	–	–	+	+	+	+
d-Glucuronic acid	–	–	–	–	–	–	+
α-hydroxy-Butyric acid	+	–	–	+	–	–	–
l-Lactic acid	+	–	–	+	–	–	–
l-Malic acid	+	–	+	+	+	+	–
Methyl pyruvate	–	–	–	+	–	–	–
l-Pyroglutamic acid	+	+	+	+	+	–	–
(d) Resistance to inhibitory compounds							
Lincomycin	+	–	–	–	–	+	–
Potassium tellurite	+	+	+	+	+	+	–
Rifamycin SV	+	+	+	+	+	+	–
Sodium chloride (4%, w/v)	+	+	+	+	+	+	–
Sodium lactate (1%)	+	+	–	+	+	+	–
Tetrazolium blue	+	–	–	–	–	+	–
Tetrazolium violet	+	–	–	–	–	+	–
Troleandomycin	+	–	–	–	–	+	–
(e) Growth at							
pH 5	+	+	–	–	+	+	–
Degradation test							
Casein	+	+	–	–	–	–	–
Growth at							
10 °C	+	+	+	+	+	+	–
45 °C	–	–	–	+	–	+	–

+ positive result; − negative result

Positive results recorded for all of the isolates and the *S. ghanaensis* type strain:API ZYM tests: acid and alkaline phosphatases, cysteine arylamidase, esterase lipase (C8), β-galactosidase, leucine and valine arylamidasesGEN III BIOLOG microplate tests: utilization of d-alanine, l-glutamic acid, l-histidine, inosine (amino acids), *N*-acetyl-d-glucosamine (amino-monosaccharide), glycyl-l-proline (dipeptide), acetic acid, acetoacetic acid, γ-amino-l-butyric acid, *p*-hydroxy-phenylacetic acid, d-malic acid, propionic acid (organic acids), gelatin (polymer), d-cellobiose, dextrin, d-fructose, d-galactose, β-gentiobiose, d-glucose, 3-*O*-methyl-d-glucose, d-maltose, d-mannose, d-melibiose, sucrose, d-trehalose, d-turanose (sugars), d-galacturonic acid, l-galacturonic acid-Ý-lactone, d-gluconic acid, β-hydroxy-butyric acid (sugar acids), d-arabitol, glycerol, *myo*-inositol (sugar alcohols), growth at pH6, resistance to aztreonam, guanidine hydrochloride, lincomycin, nalidixic acid, niaproof and growth in the presence of, sodium bromate, and sodium formate (1%, w/v)Other phenotypic tests: aesculin and arbutin hydrolysis, degradation of adenine, elastin, hypoxanthine, starch, l-tyrosine, Tweens 40, 60 and 80 and growth at 20, 30 and 40 °C Negative results recorded for all of the isolates and for the *S. ghanaensis* type strain:API ZYM tests: α-chymotrypsin, α-fucosidase, α-galactosidase, β-glucosidase, β-glucuronidase, lipase (C14) and naphthol-AS-BI-phosphohydrolaseGEN III BIOLOG microplate tests: utilization of d-aspartic acid, d-serine #1, d-serine #2 (amino acids), d-fructose-6-phosphate, stachyose (sugars), glucuronamide (amine hexose), d-lactic acid methyl ester, mucic acid, quinic acid, d-saccharic acid (organic acids), pectin (polymer) and resistance to fusidic acid and minocyclineOther phenotypic tests: allantoin and urea hydrolysis, nitrate reduction, H_2_S production, degradation of cellulose, chitin, guanine, tributyrin, uric acid, xanthine, xylan and growth in the presence of sodium chloride (8%, w/v) and at 4 and 50 °C Non-reproducible results recorded for all of the strains:GEN III BIOLOG microplate tests: utilisation of d- raffinose (trisaccharide), l-rhamnose (monosaccharide), l-alanine, l-aspartic acid (amino acids), citric acid, formic acid, α-keto-glutaric acid (organic acids), Tween 40 (surfactant); resistance to vancomycin (antibiotic), lithium chloride (heavy metal) and growth in presence of sodium butyrate (salt)

Isolates KNN35.1b^T^ and KNN35.2b were found to inhibit the growth of the wild type strains of *B. subtilis, E. coli, P. fluorescens* and *S. aureus*, whilst isolates KNN6.11a and KNN83.b only inhibited the growth of the *B. subtilis* strains; in all cases inhibition zones were extensive ranging from 13 to 24 mm.

It can be concluded that the six isolates from the hyper-arid Atacama Desert soils have identical or almost identical 16S rRNA and MLSA gene sequences and share many phenotypic features in common, some of which distinguish them from the type strain of *S. ghanaensis*, their close phylogenetic neighbour in the *Streptomyces* MLSA gene tree generated from the five housekeeping genes. It is, therefore, proposed that the isolates be recognised as a new species within the genus *Streptomyces,* named *Streptomyces asenjonii* sp. nov. It seems likely that *S. asenjonii* strains are common in hyper-arid Atacama Desert soils as additional isolates from the Salar de Atacama sampling site show the same aerial/substrate mycelial and diffusible pigment colours as the current isolates when grown on oatmeal agar. Colour groups such as these have been shown to be reliable indicators of *Streptomyces* species identity (Antony-Babu et al. 2010; Goodfellow and Fiedler 2010).

It is evident from Table 1 that the type strains of *S. ghanaensis* and *S. viridospo*rus have a low MLSA distance consistent with their assignment to a single genomic species. Indeed, in their extensive MLSA study of type strains of the family *Streptomycetaceae*, Labeda et al. (2017) noted that *S. ghanaensis* NRRL B-12104^T^ (also ATCC 14672^T^) is a later synonym of *S. viridosporus* NRRL ISP-5243^T^. This observation was confirmed by determination of the ANIm and ANIb average-nucleotide identity values between draft genome sequences of the type strains of these species using the calculate_ani.py script (https://github.com/widdowquinn/scripts/blog/master/bioinformatics/calculate_ani.py), as shown in Table S1. Note that the ANIm percentages between the genome sequences of *S. viridosporus* NRRL 2414^T^, *S. viridosporus* T7A and *S. ghanaensis* ATCC 14672^T^ are >96% and the ANIb percentages between these genomes are >97% which is indicative of species level relatedness (Richter and Rosselló-Móra 2009). Thus, according to Rule 38 of the Bacteriological Code of Nomenclature of Bacteria (Lapage et al. 1992; Parker et al. 2015), *S. viridosporus* Pridham et al. 1958 has priority over *S. ghanaensis* Wallhäuser et al. 1965. The type strains of these taxa form spiral chains of spiny to hairy spores (Kämpfer 2012), properties known to be predictive in *Streptomyces* systematics (Labeda et al. 2012) and have many physiological features in common (Kämpfer et al. 1991). Consequently, on the basis of these observations an emended description is given of *Streptomyces viridosporus* Pridham et al. (1958).

### Description of *Streptomyces asenjonii* sp. nov.

*Streptomyces asenjonii* (a.sen.jo’ni.i. N.L. gen. n., *asenjonii*, named after Juan A. Asenjo in recognition of his promotion of work on Atacama Desert actinobacteria).

Aerobic, Gram-positive actinobacteria which form an extensively branched substrate mycelium which carry aerial hyphae that differentiate into open spirals of hairy ornamented spores. Grows from 10 to 50 °C, optimally 37 °C; from pH 5 to 11, optimally 7.5; and in the presence of up to 5% w/v sodium chloride. Produces acid and alkaline phosphatase, cysteine arylamidase, esterase lipase (C8), β-galactosidase, *N*-acetyl-β-glucosaminidase and leucine and valine arylamidases (API ZYM tests), hydrolyses aesculin and arbutin, degrades adenine, elastin, hypoxanthine, starch, l–tyrosine and Tweens 20, 40, 60 and 80 and is resistant to aztreonam. Additional phenotypic properties are given in Table 4. The cell wall peptidoglycan contains *LL*-diaminopimelic acid and whole cell hydrolysates contain glucose, mannose, ribose and xylose. The major fatty acid is *iso*-hexadecanoic acid (iso-C_16:0_) and the predominant menaquinones are MK9(H_6_) and MK9(H_8_). The polar lipid profile contains diphosphatidylglycerol, glycophospholipid, phosphatidylethanolamine, phosphatidylglycerol and phosphatidylinositol.

The type strain KNN 35.1b^T^ (NCIMB 15082^T^ = NRRL B-65050^T^) and strains KNN 6.11a, KNN 35.2b, KNN 42.f, KNN 48.3e and KNN 83.e were isolated from hyper-arid Atacama Desert soils. The GenBank/EMBL/DDBJ accession number for the 16S rRNA gene sequence of strain KNN 35.1b^T^ is LT621750. The Digital Protologue database TaxoNumber for strain KNN 35.1b^T^ is TA00093.

### Emended description of *Streptomyces viridosporus* Pridham, Hesseltine and Benedict 1958, 67^AL^

#### Heterotypic synonym: *Streptomyces ghanaensis* Wallhäusser, Nesemann, Präve and Steigler 1966, 734^AL^

Most of the data are taken from Kämpfer et al. (1991) and Kämpfer (2012).

Aerobic, Gram-stain positive actinobacteria that form substrate mycelia which bear a green aerial spore mass on glycerol-asparagine, salts-starch, oatmeal and yeast extract-malt extract agars. Short chains of over 10 spores are formed on these media. Spore surfaces are spiny to hairy. Melanoid pigments are not formed on peptone-yeast extract-iron or tyrosine agar or in tryptone-yeast broth. l-arabinose, d-cellobiose, d-fructose, d-galactose, d-glucose, d- glucosamine, glycogen, d-maltose, d-mannitol, d-mannose, starch, d-trehalose, d-xylose, acetate, fumarate, and pyruvate are used as sole carbon sources, but not *myo*-inositol, d-tagatose or d-turanose.

The source of the type strain NRRL ISP-5243^T^ = NRRL 2414^T^ is not known; ‘*S. ghanaensis*’ NRRL B-12104 was isolated from soil from Ghana.

The type strain is ATCC 27479 = CBS 654.72 = BCRC (formerly CCRC) 11870 = CCUG 37512 = DSM 40243 = NBRC 13353 = IMET 43514 = JCM 4859 = KCTC 9145 = NCIMB 9824 = NRRL 2414 = NRRL-ISP 5243 = RIA 1314 = VKM Ac-1769 = VKM Ac-618.

## Electronic supplementary material

Below is the link to the electronic supplementary material.

Supplementary material 1 (DOCX 169 kb)
