# Supplementary material for: Streptomyces asenjonii sp. nov., isolated from hyper-arid Atacama Desert soils and emended description of Streptomyces viridosporus Pridham et al. 1958
Source: Antonie Van Leeuwenhoek. 2017 Jun 6;110(9):1133–48. doi: 10.1007/s10482-017-0886-7 (PMC5559561; doi:10.1007/s10482-017-0886-7)
Supplement: Supplementary file 1 — Supplementary material 1 (DOCX 169 kb) [file 10482_2017_886_MOESM1_ESM.docx]

Table S1 Average nucleotide identities between *Streptomyces* genome sequences.

ANI percentages calculated using the calculate_ani.py script (<https://github.com/widdowquinn/sripts/blob/master/bioinformatics/calculate_ani.py>) which implements the methods described by Goris et al (2007) and Richter et al (2009). The results determined with the ANIm method are shown on the lower left and the upper right values represent ANI percentages calculated using the ANIb method.

|  | NRRL 2414^T^ | T7A | ATCC 14672^T^ | NRRL B-2713^T^ | NRRL B-3040 |
| --- | --- | --- | --- | --- | --- |
| *Streptomyces viridosporus* NRRL 2414^T^  (MSGP00000000) |  | **97.31** | **97.32** | 88.93 | 88.87 |
| *Streptomyces viridosporus* T7A  (AJFD000000000) | **96.91** |  | **99.11** | 88.70 | 88.65 |
| *Streptomyces ghanaensis* ATCC 14672^T^  (ABYA00000000) | **96.92** | **99.15** |  | 88.72 | 88.63 |
| *Streptomyces hirsutus* NRRL B-2713^T^  (LIQT00000000) | 86.55 | 86.27 | 86.47 |  | **97.79** |
| *Streptomyces cyanoalbus* NRRL B-3040^T^ (LIPS00000000) | 86.41 | 86.12 | 86.17 | **97.62** |  |

Figure S1 Two-dimensional thin-layer chromatography of polar lipids of *Streptomyces* isolates KNN 35.1b, KNN 35.2b^T^ and *S. ghanaensis* stained with molybdatophosphoric acid- spray (Sigma P1518). Key: DPG, diphosphatidylglycerol; PG, phosphatidylglycerol; PI, phosphatidylinositol; PE, phosphatidylethanolamine; AL, aminolipid, GPL glycophospholipid, PL, phospholipid and L, unknown lipids.
